# Everett Mendelsohn: A Splendid Mentor, Primary Source, and Champion

**DOI:** 10.1007/s10739-023-09749-1

**Published:** 2023-12-13

**Authors:** Rena Selya

**Affiliations:** Cedars-Sinai Medical Center, Los Angeles, CA USA

Everett’s death this summer was a blow that brought back a slew of powerful memories. He was a vital part of my graduate experience at Harvard, from my interview with him on a cold snowy day in January 1996 to when I walked across the stage into his proud embrace after I received my PhD in June of 2002. In between, he was a guide, a mentor, a primary source, and a friend.

I started graduate school with a general interest in 20th century biology and so I was pleased when Everett offered a history of biology course in the spring of my first year. When it came time to choose a research paper topic, I went to Everett’s office hours to see what he thought about my writing a paper on James Watson, who both fascinated and repelled me. Everett said, “If you're interested in Watson why don't you read my friend Salvador Luria’s autobiography (Luria 1984) and see what he said about his first graduate student?” Everett and Luria had met in the early 1960s when they were both involved in the anti-Vietnam War movement in Boston, and they remained close colleagues and friends until Luria's death in 1991. I dutifully checked the book out of the library and was instantly charmed by Luria’s candor about his scientific career as well as his commitment to political activism in the United States. So a few days later came back to Everett with another idea. What if I wrote my dissertation on Salvador Luria? Everett's face lit up as he proclaimed it a “splendid” idea. He helped me locate Luria’s papers at the American Philosophical Society Library and wrote a glowing letter in support of a graduate fellowship to start my research.

Through his personal connection to Luria, Everett became not only my dissertation advisor, but also a primary source. He generously gave me access to his papers in the Harvard archive, so that I could reconstruct Luria’s involvement in the Boston Area Faculty Group on Public Issues. This informal group, also known by its unwieldy acronym BAFGOPI, was an organization of professors and researchers, including Everett, who were united by their commitment to “the preservation of peace with freedom and the prevention of war.” They organized letter writing campaigns, published op-eds, took out full page ads in the *New York Times* protesting the war, and mobilized academics to actively protest American involvement in Vietnam. In 1967 they put out a call to convene a Congress of American professors, reminding their colleagues that

as university professors we have unique skills. We also bear special responsibilities. Today our great responsibility is to help restore the vigor of democratic institutions in our land…. To meet the responsibility, we must use our unmatched knowledge and expertise to formulate and diagnose policy alternatives. We must use our knowledge to act on public issues. We can do much in our own campuses and communities. Through coordinating our efforts we will add weight and effectiveness to local action.^1^

Everett threw his lot in with the scientific community and put that responsibility to full use in 1972 when he was a vice president of the American Association for the Advancement of Science. He organized a committee that included Luria, George Wald, and Albert Szent-Gyorgyi to mobilize their scientific colleagues to commit themselves publicly to the ideal of what they called “Science in the Service of Life.” While only 250 AAAS members signed their draft letter to President Nixon and only 100 attended their rally in Washington DC on December 28, Everett nevertheless successfully introduced “an emergency motion” at the AAAS Council meeting on December 30th, 1972. “In an unprecedented expression of political sentiment,” the Council adopted a “bluntly phrased resolution” condemning the war. The statement combined scientific and political sensibilities in an unequivocal stance against the current government policy.

As scientists we cannot remain silent while the richest and most powerful nation of the twentieth century uses the resources of modern science to intervene in the problems of poor and distant lands. Our Association objective, “To increase public understanding and appreciation of the importance and promise of the methods of science in human progress” compels us to refute the view that scientists and engineers are responsible for and endorse, by their actions or by their silent consent, the wanton destruction of man and environment, in this case through warfare. (Lyons 1972; Gillette 1973a, 1973b)

When I presented this episode in a paper about Luria’s political activism at my first ever History of Science Society meeting in November 2001, Everett chimed in and recalled that he was able to slip the motion in after the Council had approved a motion condemning the US Army for using live horses to do research on the damage done by land mines. If they had spoken out to protect the horses, then of course they would support a resolution protecting people and the environment!

I wrote the dissertation chapter about Luria’s (and Everett’s) political activities around the Vietnam War in the chaotic and depressing weeks after September 11th 2001. I remember speaking with Everett about how the academic community seemed so much less engaged in the public conversations around the appropriate response to the terror attacks than he and his peers had been in the 1960s and 70s. We also had numerous conversations about his ongoing participation in Middle East peace endeavors and while we didn't always agree about the details, I admired his faith and optimism that peace through negotiation was possible. He was always solicitous and sympathetic about my worry about my family members in Israel. I'm writing this memorial essay in the second week of October 2023, and I can only imagine how heartbroken Everett would be to know about the terrible violence that has transpired in Israel and Gaza this week.

Everett was a role model for graduate students as we navigated the world of academic socializing and scholarly interaction. Every year one nervous new student would ask him, “Professor Mendelsohn, where are you from?” to which Everett would cheerfully reply, “The Bronx!” He was a master of the art of asking good questions and he knew a little bit about almost every aspect of the history of science, so he was always able to engage with students and peers in a meaningful way. Whether in a seminar or a colloquium, Everett always managed to ask a respectful question that would not only indicate that he had been listening carefully but also gave the speaker an opportunity to develop their ideas or express themselves more fully. He was usually the first one to raise his hand, summarizing the argument and sparking lively conversations.

For many years, Everett co-taught the introductory methods course for incoming graduate students in the history of science. He would methodically and carefully walk us through the historiography of our field as well as the various theoretical schools that scholars would draw upon. His support was financial as well as intellectual. Everett had an uncanny ability to find untapped pots of Harvard money for graduate students whose funding had fallen through, and made sure that we had enough support to travel to archives and conferences. Everett was also deeply invested in our personal development and successes. He delighted in the news of our weddings and babies, and often sought points of personal connection. Even though he did not practice Judaism, he never missed an opportunity to tell me his favorite Yiddish joke around the Jewish holidays. (“What do you say to the Pope on Passover? Good Yontif, Pontiff!”). On a more practical level, I deeply appreciated his efforts to use a kosher caterer for all the food at History of Science Department events so that I and other Jewish students would not feel left out or isolated.

It was a treat to watch Everett lecture. I had the opportunity to serve as the head teaching fellow for his “Historical Studies B46: The Darwinian Revolution” core course in the spring of 2000. Everett captivated the 200 undergraduates who were sitting in that lecture hall, deftly weaving anecdotes about Charles Darwin into a larger framework about the intellectual, social, and political implications of the theory of evolution by natural selection. To this day, whenever I teach about Darwin, I hear Everett’s voice in my head, emphasizing that the *Beagle* was a small boat. Everett was generous to a fault with his undergraduate students. After a long night of grading final exams and collating blue books, Everett turned to me and said, “OK so the students who got the lowest scores will get C, and then we can curve the grades up from there.” When I protested that students who clearly had not grasped the material would nevertheless pass the class, he offered me a compromise: those students would get a C minus. That was as far as he was willing to go.

It took more than twenty years for me to publish my book about Luria. Everett was too frail to attend the publication celebration at MIT last October but a few months later I got a wonderful e-mail from him after he had read the book. He wrote in all caps, “SALVA LIVES!!!” and thanked me for creating an accurate portrait of the man he had known. In his message, he lamented that at 91 and after several strokes, “my opportunity for new ventures is limited,” but tried to put a positive spin on it: “More time for the *NYTimes* and other books and articles that catch my fancy.” He was curious and engaged to the very end and I'm grateful to have had the opportunity to remind him of the political engagement he so enthusiastically and passionately participated in more than 50 years ago. Everett was a scholar, a teacher, an activist, and a mensch. May his memory and example be a blessing for us all.

## Footnotes

1 BAFGOPI Folder, Everett Mendelsohn Papers, Harvard University Archives.
